# Dr. Midelton on Treatment by Continuous Counter-Irritation

**Published:** 1914-01-31

**Authors:** W. J. Midelton

**Affiliations:** Bournemouth


### THE EDITOR'S LETTER-BOX.

### Dr. Midelton on Treatment by Continuous Counter-Irritation.

*To the Editor of* The Hospital.

Sir,—Referring to the paragraph on my results with continuous counter-irritation in rheumatoid arthritis in your issue of January 17, 1914, I should like to give you some further experiences of this method.

From the earliest times counter-irritation in one form or another has been utilised in the treatment of human ailments. The agencies employed in carrying out this procedure are, of course, various. One of the most universal and readily obtainable is the heat of the sun; next in order, probably, comes hot water. Then we have mustard, capsicum, croton oil, cantharides, the actual cautery, and so forth.

For more than thirteen years I have employed the blister followed by the application of savin ointment to the raw surfaces produced. I have found this plan particularly successful in the treatment of chronic non-tubercular arthritis, but as its action is "general," I have also found it useful in paralysis in which the spinal cord is chiefly affected. For more than five years I have employed acupuncture and irritants with a view to producing a pustular eruption. This is also a remedy of the "all-round" order, and I am very pleased with it. I claim excellent results in skin diseases, arthritis, paralysis, etc.

Another form of great value is the galvano-cautery. I have used this chiefly in asthma and neuritis, but it can be employed with advantage in other ailments. In order to produce the best results these measures must be perseveringly carried out for long periods, and it must be obvious to anyone that experience in their use is highly essential. Carefully dealt with they are free from risk, and the discomfort to the patient is so slight that anyone who will not put up with it deserves to go on suffering. They have greatly benefited patients who have suffered for years and found very little relief from other remedies.

I have often been asked as to how this method acts, and have to admit that there is no really satisfactory explanation at present available. However, I have arrived at certain conclusions as the result of many years' clinical experience and observation, probably unique. As far as I know, there is no one who has practised these methods as extensively as I have, taking them collectively, and particularly the blister followed by savin ointment. One of my arthritis patients has been under my close observation for over thirteen years. She responded remarkably to treatment, and notes of her case have been published. Many of my patients reside near me, and this gives me an advantage not possessed by many practitioners, especially some of the leading teachers of medicine in London or elsewhere.

In the case of the blister followed by savin ointment, my applications have been made in the immediate neighbourhood of the cervical and lumbar enlargements of the spinal cord, and with strikingly beneficial effects. In the hands of an expert who has learnt when he may do so with safety, a large blister may be used with great advantage where a small one would fail. I have personally had no opportunity of using blisters in cases of true insanity as it is generally understood, but in borderland conditions the effect has sometimes been most gratifying. In so-called functional conditions also one meets with success; this includes disturbance of functions of special organs, such as nose, eyes, ears, etc.

A gentleman sent for me complaining of loss of visual power to such an extent that he could not read. A prompt application of acupuncture with irritants over a large area on the upper part of the back, extending on to the lower part of the neck, was followed by complete restoration of sight within twenty-four hours. Another of my patients, suffering from chronic arthritis, with great loss of power and muscular wasting, was able to put her hands above her head within three days of the application of two large blisters, one on each side of the spine, at the base of the neck and upper dorsal region. Within six weeks she was hard at work, and has never had a day's real illness since or missed a day's work of necessity. The treatment was carried out over ten years ago. I argue that here there was congestion in the lower part of the brain and upper part of the spinal cord interfering with the proper transition of impulses from the brain to the muscles, etc., and that the blisters relieved this.

One great advantage of these measures is that although they can be depended on to act in a most beneficial manner alone, they do not preclude the employment of suitable drugs or other remedies—quite the contrary.

Faithfully yours,
W. J. Midelton.

Bournemouth,
January 18, 1914.

